# Early adequate empiric antifungal therapy and 28-day mortality in ICU patients with candidemia: a pooled patient-level analysis of EUROBACT-1 and EUROBACT-2 cohorts

**DOI:** 10.1186/s13054-026-06373-3

**Published:** 2026-10-16

**Authors:** Hannah Wozniak, Alexis Tabah, Matteo Bassetti, Kevin Laupland, José Artur Paiva, Stéphane Ruckly, François Barbier, Jean-François Timsit, Niccolò Buetti, EUROBACT-1 Study Group, Fatemeh Aalinezhad, Heleen Aardema, Eman Abdulwahed, Fayez Abilama, Fathi A. Abousnina, Fekri Abroug, José Oscar Acevedo, Tayfun Adanir, Fowotade Adeola, Canan Agalar, Candy Garcia Aguilar, Fatemeh Ahmadi, Abdullah Khudhur Ahmed, Samiul Ahmed, Halis Akalin, Türkay Akbas, Ozlem O. Akdogan, Joyce Åkerlund, I. Ozkan Akinci, Murat Akova, Firdevs Aksoy, Sabahat Cagan Aktas, Ahmet Akyol, Amer Aldhalia, Serge Alfandari, Almajdoub Ali Mohammed Ali, Fadi Ali, Saedah Ali, Ahmed Aljabbary, Ghaleb A. Almekhlafi, Bushray Almiqlash, Mariota Panagiota Almiroudi, Irene Alonzo, Adel Alsisi, Nilgün Altin, Neriman Defne Altintas, Adrian Davalos-Alvarez, Madalena Alves, Joaquin Amador Amerigo, Fereshteh Amiri, Sarah Amro, Anastasia Anderzhanova, Hollie Angell, Djillali Annane, Sylvain Anselme, David Antcliffe, Georgios Anthopoulos, Massimo Antonelli, Eleni Antoniadou, Supriya Antrolikar, Elli Antypa, Christelle Aparicio, Marta Aparicio, Yutthana Apichatbutr, Ahmed Aqeelah, Charlotte Arbelot, Nerea Lopez De Arbina, Laurent Argaud, Sousa Sonia Arias, Apostolos Armaganidis, Fernando Armestar, Maria Pilar Gracia Arnillas, Kostoula Arvaniti, Mj Asensio, Gabriel Asiyanbi, Abdullah Tarik Aslan, Zoi Athanasa, Kaori Atobe, Antony George Attokaran, Ben Attwood, Johann Auchabie, Ahmed K. Awad, Maher Awad, Samia Ayed, J. Ignacio Ayestaran, Esra Aykac, Abd. Hamid Azman, Elie Azoulay, Baurzhan Babashev, Sailaja K. Baby, Gaëtan Badre, Reza Baghbanian, Nadia Baig, Rao Baikady, Manan Bajaj, Stuart Baker, Lubna Bakr, Dorota Banach, François Barbier, Arvind Baronia, Mina Barsoum, Anabela Bártolo, Ayse Batirel, Uros Batranovic, Denise Battaglini, Thomas Baudry, Tom Baumer, Yasar Bayindir, Nurcan N. Baykam, Yelena Bazhykayeva, Vasileios Bekos, Z. Belkhadir, Davide Bellina, Daniel Belman, Vladislav Belskiy, Renata Beranger, Dennis Bergmans, Rosana Munoz Bermudez, Silvia Bernreiter, Marielle Boyer-Besseyre, M. Beuzelin, Neeraj Bhadange, Yeliz Bilir, Hala Bilkhayr, Vishwanath Biradar, Alessandra Bisanti, Patrick Biston, E. Black, Marine Rossi-Blancher, Armando Blanco, Frank Bloos, Stijn Blot, Lajos Bogar, Jozef Bojko, Marc J. M. Bonten, Seyed Hamid Borsi, Perrine Bortolotti, Payel Bose, Isabel Botelho, Mounir Bouaziz, Charra Boubaker, Ahmad Bouhuwaish, Brahim Boukatta, Jérémy Bourenne, Marc Bourgeois, Hendrik Bracht, Ana Vujaklija Brajkovic, David Brewster, Gleyse Brigitte, Hanna Broda, Cédric Bruel, Ahmad Buimsaedah, Christian Brun-Buisson, Yaroslava Burma, Liesbet De Bus, Unase Buyukkocak, Nahit Çakar, Rumeysa Cakmak, Silvia Calvino, Juan Ramón Cortés Cañones, Teresa Cardoso, Simone Carelli, Jean Carlet, Nuno Cartoze, Alexandre Carvalho, Frederico Bruzzi de Carvalho, Vanessa Casares, Eliana Caser, Jérémy Castanera, Paula Castelões, Roberto Alejandro Castillo, Beatriz Catalan, Pablo Centeno, Jaroslav Cerman, Solakoglu Ceyhun, Mohammad Karam Chaaban, Jagadish Chandran, Vasiliki Chantziara, Julien Charpentier, Marinos Chatzis, Xiaomei Chen, Jose G. Dominguez-Cherit, Ronco Chiara, Jean-Daniel Chiche, Supattra Chiewroongroj, Moses Chikungwa, Robert Choma, Nai-Kuan Chou, Georgios Choutas, Achilleas Chovas, Herault Marie-Christine, Christos Christopoulos, Maria Chukina, Cong-Tat Cia, Giorgia Citterio, Katherine Cobb, Cristian Cobilinschi, Onur Çolak, Sait Çolak, Stephen Cole, Andrea Cortegiani, Ahmet Cosar, Tito da Costa, Vasco Costa, Antonella Cotoia, Elisabeth Coupez, Marco Covotta, Amanda Cowton, Melanie Cree, Akos Csomos, Maricarmen Gilavert Cuevas, Rui Pedro Cunha, Aida Custovic, Gholamreza Dabiri, Ivan Daroui, Avijatri Datta, Alexandre Debrumetz, Johan Decruyenaere, Vesna Degoricija, Jean Philippe Delabre, Sophie Demeret, Emine Kubra Dindar Demiray, Tarek Dendane, Wouter Denys, Pieter Depuydt, Ibironke Desalu, Armand Mekonted Dessap, Adei Diab, Celeste Dias, Maria Conceição Dias, Ahmet Dilek, Georges Dimopoulos, Flora Djanikian, Rachael Dolan, Iwona Dragan, Carol Dsilva, Meili Duan, Tiago Duarte, Simon Dubler, Thibault Duburcq, Thierry Dulac, Matthieu Duprey, Michel Durand, Jyotsna Dwivedi, Alicia Edin, Anna Efthymiou, Philippe Eggimann, Merihan A. Elbadawy, Mohamed Elbahnasawy, Abdurahaman Elbuzidi, Amr Elhadidy, Omar Elmandouh, Omar Elrabi, Ahmed Elsayed, Ari Ercole, Renaud Lepaul Ercole, Cem Erdogan, Aleksandr Eremenko, Ahmet Eroglu, Ege Eroglu, Çigdem Erol, Yasemin Ersoy, Gulden Ersoz, Etienne Escudier, Lorena Forcelledo Espina, Zollei Eva, Guliz Evik, Sebastien Faivre, Jansen Falcão, Aya Fares, Muriel Fartoukh, Maria Faulkner, Thomas Felbinger, Rubén Fernández, Alcina Ferreira, Leonardo Ferreira, Ricard Ferrer-Roca, Richard Ferrer, António Manuel Pereira de Figueiredo, Georgios Fildisis, Daniela Filipescu, Daniela Carmen Filipescu, Mackenzie Finnis, Silvia Fiorelli, Claire Dahyot-Fizelier, Matias Flores, Kean Khang Fong, Arnaud Foucrier, Cheryl Fourie, Carlos França, Voillet Francois, Uwe Franke, Shigeki Fujitani, Mikhail Furman, Csóka Gábor, Denis Gaigolnik, Marc Gainnier, Alberto Orejas Gallego, Marwa Gamra, Pierre Garcon, Pau Garro, Alexandra Gavala, Srdjan Gavrilovic, Lina De Geer, Nan Geng, Christos Georgiadis, Dimitris Georgopoulos, Maria Georgopoulou, Nikoletta Gritsi-Gerogianni, A. Ghannam, Pralay Ghosh, Daniele Roberto Giacobbe, Maria Giannakoy, Sebastien Gibot, Nermin Kelebek Girgin, Eleni Gkeka, Ken Junyang Goh, Qing Yuan Goh, Senay Goksu, Ugur Goktas, Gemma Gomà, Juan P. Romero-Gonzalez, Ivan Gornik, Thomas Gottlieb, Antje Gottschalk, Shraddha Goyal, Maria Pilar Gracia, Clive Graham, Angelo Gratarola, Valentina Di Gravio, Ina Filipovic-Grcic, Ioana Grigoras, Ioana Marina Grintescu, Alexey Gritsan, Iwona Pietraszek-Grzywaczewska, Andrea Di Guardo, Loïc Le Guennec, Charlotte Guesdon, Nádia Guimarães, Kursat Gundogan, Yunus Gürbüz, Mohan Gurjar, Seda Guzeldag, Abdelhamid Hachimi, Lionel Haentjens, Qiu Haibo, Daniel Hall, Hytham K. S. Hamid, Michael Haney, Belal Harara, Hayat Ben Hasan, M. Shahnaz Hasan, Rabab Shaban Ben Hasan, Rajib Hasan, Satoru Hashimoto, Yoshiro Hayashi, Aisha Khaled Hdada, Alexandra Heininger, Hanan M. Hemead, Pierre Henin, Mariana Hermosillo, Mara Ocotlan Gonzalez Herrera, Tayne Hewer, Sandra Hoheisen, Isabelle Hollevoet, Ahmed Gaber Sadik Homaidan, Koji Hosokawa, Ashraf El Houfi, Brahim Housni, Ali Ait Hssain, Chungfang Hu, Ting-Yu Hu, Lu Wei-Hua, Min Huang, Brigitte Tejeda-Huezo, Keith Hugill, Sally K. Humphreys, Shihan Mahmud Redwanul Huq, Luis Sánchez-Hurtado, A. Husain, Cristina Iasonidou, Rebecca Ibrahim, Olusola Idowu, Nadia Imzi, Josu Insausti, Aikaterini Ioakeimidou, Mariachiara Ippolito, Mohammad Motiul Islam, Matthew Jackson, Mohammad Al-Jadaan, Nikhilesh Jain, Stephan Jakob, Philippe Jamaer, Samir Jamali, Muhamad Fadhil Hadi Jamaluddin, Sylvie Janny, C’havala Jaramillo, Fatemeh Javaherforoosh, Mustafa Abu Jayyab, Kyeongman Jeon, S. Jhanji, Xie Jianfeng, Rania Jobran, Aimee Johnson, Antonio Fagundes Jr, Cartier Julien, Ilya Kagan, Evangelos Kaimakamis, Ayse Kaya Kalem, Pierre Kalfon, Zhannur Kaligozhin, Hatem Kallel, C. Kampolis, Nabil Kanjaa, Santhana Kannan, Ridvan Karaali, Andreas Karabinis, Ilias Karaiskos, Ali Adil Ali Karar, Edin Karic, Saurabh Karmakar, Toshiyuki Karumai, Davood Kashipazha, Ismail Kati, Hideaki Kato, Chrysostomos Katsenos, Bircan Kayaaslan, Allison Keegan, Melanie Kent, Reena Khade, Arash Kiani, Eric Kipnis, Tamas Kiss, Maria Klimathianaki, Kada Klouche, Sesin Kocagoz, Evangelos Kogkopoulos, Iftihar Koksal, Apostolos Komnos, Irmina Kaptur Komorowska, Wladyslaw Koscielniak, Wlodzimierz Kostyrka, Nikhil Kothari, Anna Kotkowska, Achille Kouatchet, Despoina Koulenti, Vasilios Koulouras, Katerina Kounougeri, Vasilios Kouroulas, Anastasia Koutsikou, Antonia Koutsoukou, Vasiliki Koutsoukou, Pedja Kovacevic, Nóra Ágota Kovács, Peter Kraffft, Wolfgang A. Krueger, Andriy Krystopchuk, Shih-Chi Ku, Li-Kuo Kuo, Akira Kuriyama, Ibrahim Kurt, Hulya Kusoglu, Demetrios J. Kutsogiannis, Andrea Lay Hoon Kwa, Christina Kydona, Magdalini Kyriakopoulou, Alexandra Lahana, Marcus Lance, Romaric Larcher, Hrvoje Lasic, Markus Laube, Anne Launoy, Kevin B. Laupland, Nathalie Layos, Jesús Nicolás Pantoja Leal, Hervé Lebbinck, Anne Leditschke, Sara Ledo, Anne Ledtischke, Sang-Min Lee, Jean-Yves Lefrant, James Lennard, Marc Leone, Philippe Lepper, Francisco Alvarez-Lerma, Olivier Leroy, Henry Lessire, Phillip Levin, Andrew Yunkai Li, Adamantia Liapikou, Sui An Lie, Mun Thing Liew, Deng Lijing, Jia Yan Lim, Sung Yoon Lim, Jeffrey Lipman, Edward Litton, Giovanni Lodi, Ignacio Martin-Loeches, S. Loftus, Rebecca Longbottom, Patricia Mabel Lopez, Guillaume Louis, Francisco Lucena, Marcelo Lugarinho, Olguta Lungu, Jili Luo, Xiaochun Ma, Caroline Macfie, Felipe Carrhá Machado, Eleni Magira, Marat Magomedov, Ata Mahmoodpoor, S. Abed-Maillard, Jun Makino, Polychronis Malliotakis, Kostas Mandragos, Valerio Mangani, A. Marchalot, Alexandra Marcu, Ben Margetts, Kyriaki Marmanidou, Diamantina Marouli, Ana J. Marques, Ana Josefina Pinheiro Marques, Lara Marqués, Nicholas Marsden, Iñigo Martija, Claude Martin, Maria Martinez, Salomé Martins, Gernot Marx, Emilio Maseda, Mansoor Masjedi, Alexandre Massri, Dimitrios Matamis, Mariana Kristina Matei, Philippe Mateu, Takeo Matsuyoshi, Edward Maul, Virginie Maxime, Mohd Zulfakar Mazlan, Lorena McCoshen, Peter Mcguigan, Rachel Mckee, Bruno Mégarbane, Zahra Mehraban, Selcuk Mehtap, Yulia Meleshkina, António Carvalho Mendes, Sybille Merceron, Yevgeniy Merenkov, Jean-Baptiste Mesland, Carlos Metz, Anthony Le Meur, Jason Meyer, Martha Michalia, Borbala Mikos, Adam Mikstacki, David Milliss, Chen Mingmin, Jean-Paul Mira, Michele Mirabella, Isabel Miranda, Yuka Mishima, Krushna Chandra Misra, Benoit Misset, Toshiki Mizobe, Leora Moldovan, Elena Molinos, Phyllis Clouva-Molyvdas, Tomas Mon, Pablo Montefiore, Luca Montini, Luca Maria Montini, P. Montravers, Giorgia Montrucchio, P. Dhakshina Moorthi, Andrew Conway Morris, Natalia Morvillo, Tarek Mostafa, Said Motaouakkil, Doumiri Mouhssine, José Pedro Moura, Miras Mugazov, Sudipta Mukherjee, Randeep Mulhi, Syed Nabeel Muzaffar, Pavlos Myrianthefs, Rashid Nadeem, Jacek Nadolski, Khalid Mahmood Khan Nafees, Michio Nagashima, Georgios Nakos, Christodoulos Nathanail, Miguel Angel Blasco-Navalpotro, Kangqi Ng, Louis Xiang Long Ng, Shin Yi Ng, Alexandru Nica, Orlando R. Perez-Nieto, Ilona Nikolaidou, Chara Nikolaou, Charikleia Nikolaou, Mario Nikolic, Janardan Nimbolkar, Luo Ning, Shaikh Nissar, Aikaterini Nodarou, Cindérella Noel, Zuraini Md Noor, Mariana Novo, Tomasz Nowakowski, Yanina Nuccetelli, Cristina Nunes, Marina Oikonomou, Motunrayo Oladimeji, John O. Olatosi, Mariana Oliveira, Koichiro Omori, Caroline Yu Ming Ong, Evelien A. N. Oostdijk, Godelive Opdenacker, Maité Garrouste-Orgeas, Nermin Osman, Islem Ouanes, Maryam Ouhadian, Ahmad Ozair, Dilek Özcengiz, Cansu Ozgen, Barcin Ozturk, Jean-Luc Pagani, Jl Pagani, Madhumati Pahuja, José Artur Paiva, Olivier Pajot, Anna Maria Palermo, Eirini Papageorgiou, M. Papanikolaou, Spiros Papanikolaou, Georgios Papathanakos, Sofia Papoti, Theoniki Paraforou, Benjamin J. Parcell, Seung Yong Park, Sunghoon Park, Gennaro De Pascale, Dulce Pascoalinho, Bastian Pasieka, Haralambos Paskalis, Harry Paskalis, Jamie Patel, Mayur Patel, Juliette Patrier, Rémi Paulet, Mariusz Peichota, Li Peije, Lorella Pelagalli, Paolo Pelosi, Énio Pereira, José Manuel Pereira, Juliana Pereira, Tiago Pereira, Pierre-François Perrigault, Jasminka Persec, David Petitpas, Sirak Petros, Tai Pham, Lee See Pheng, François Philippart, Pawel Pietraszek, Ana Rios Pinto, Claudio Piras, Arash Pirat, Gaetan Plantefeve, Paraskevi Plantza, Ioannis Pneumatikos, Pavlos Polakis, Hamish Pollock, Mihai Popescu, Elisa Porcile, Josef Prazak, Cornelia Elena Predoi, Athanasios Prekates, Emilio Garcia Prieto, Vasiliki Psallida, Chuanyun Qian, Ke-Jian Qian, Bingyu Qin, Jessica Lishan Quah, Li Jin-Quan, Joana Queiró, María Dolores Quesada, Raihan Rabbani, Radovan Radonic, Laura Raguer, Hussain Al Rahma, Hamizah Bt Abdul Rahman, Ashok Raj, Mahesh Ramanan, Nurhikmahtul Aqilah Haji Abd Rashid, Mahboobeh Rashidi, Darshana Rathod, Keyvan Razazi, Adrian Regli, Livia Reis, Jordi Rello, Paul Jean Renaud, Foteini Renta, M. K. Renuka, Ioannis Retselas, Luis Felipe Reyes, Alejandro Suarez De La Rica, Merav Rigler, Hiroshi Rinka, Jenny Ritzema, Anna Chiara Rizzo, Brigit Roberts, Monica Rocco, Helen Rodgers, Fernando Rodriguez, Fernando Armestar-Rodriguez, M. Rodriguez, Claire Roger, Regina Roig, Roceld Rojo, Vasiliki Romanou, Ashraf Roshdy, Jérémy Rosman, Madeleine Rothen, Matthew Routledge, Christina Routsi, Noor Ahmedi Rubina, Daniel Thomas-Rueddel, Ahmed Saber, Golnar Sabetian, Motaouakkil Said, Elfayadh Saidahmed, Hiroki Saito, Aikaterini Sakagianni, Sanja Sakan, Anastasios Sakkalis, Gabriele Sales, Maria Salgado, Hend Sallam, Jorge Salluh, Nese Saltoglu, Pratit Samdani, Susana Sancho, Amanda Sanderson, Eduard Mesalles-Sanjuán, Emilio Diaz Santos, Lurdes Santos, Kemal Tolga Saracoglu, Ali Sari, Vineet Sarode, Hakimeh Sarshad, Benjamine Sarton, Mohsen Savaie, Manoj Saxena, Natalia Abu-Sayf, Maria Grazia Schlevenin, Karsten Schmidt, Rik Schoemakers, Torsten Schroeder, Nicolas De Schryver, Marcus Schultz, Rachid Seddiki, Philippe Seguin, Matías Segura, Walid Sellami, Atilla Sencan, Nicolas Serck, Issam Serghini, Simay Serin, Dimitrios Sfyras, Ankur Sharma, Priya Sharma, Saurav Shekhar, Kaladhar Sheshala, Hidenobu Shigemitsu, Shidasp Siami, Suhail Sarwar Siddiqui, Silvio A. Ñamendys-Silva, Stein Silva, Pierre Singer, Ritu Singh, Josep Sirvent, Fredrik Sjövall, Wendy Sligl, Tim Smith, Roland Smonig, Piotr Smuszkiewicz, Dimitrios Smyrniotis, Natalie Soar, Marcio Soares, Anton Sokhan, Marina Soltan, Farhad Soltani, Mathieu Le Souhaitier, Vasiliki Soulountsi, Bertrand Souweine, Dzana Spahic, Kyriaki Spiropoulou, Anna Spring, Dhadappa Damodar Sridharan, Guido Stefania, Shah Sudhirchandra, Hulya Sungurtekin, Carl Johan Svensson, Tamas Szakmany, Anna Szczesniak, Alexis Tabah, Seyed Mohammad Nasirodin Tabatabaei, Ahmed Sa Taher, Ramin Tajvidi, Shunsuke Takaki, Leyla Talan, Barbara Tamowicz, Kate Tatham, Benoit Tavernier, Murat Telli, Luís Telo, Talgat Temirov, Maie Templeton, Manuel Lomeli-Teran, Ana Teresa, Vasiliki Theodorou, Lherm Thierry, Patricia Thompson, Martial Thyrault, Rebecca E. Tilley, Jean-François Timsit, Arif Timuroglu, George Tinaslanidis, Kate Tizard, Ernesto Deloya Tomas, Dana Tomescu, Lee Kok Tong, Tomasz Torlinski, Ana Tornada, Valentina Della Torre, Marc Tran, Maksim Tribulev, Christos Tsakalakis, Pirros Tsakas, Giorgio Tulli, Burcu Tunay, Yeliz Irem Tunçel, Serban Bubenek-Turconi, Olfa Turki, Steven Tyler, Nikolas Tzovaras, Alejandro Ubeda, Euphemia Mgbosoro Ugwu, Süheyla Ünver, R. Vaidyanathan, Liana Valeanu, Andreas Valentin, Jordi Valles, Sofie F. M. Vanderhaeghen, Joel Vargas, Maria Vasileiou, Annelou Van Der Veen, György Velkey, Paula Vera, Aurélien Vesin, Dmitriy Viderman, Missael Vladimir Espinoza Villafuerte, Tixier Vincent, Natala Vistovskaya, Sheshendrasurian Visvalingam, Eszter Vitális, Ingo Voigt, Guillaume Voiriot, Efstratia Vrettou, Elisabeth De Waele, Jan De Waele, Maazouzi Ahmed Wajdi, Haji Adi Muhamad Ibnu Walid, James Walsham, Xian Yao Wan, Hao Wang, Jinfeng Wang, Ruilan Wang, Yan Wang, Dominika Wasowska, Elizabeth Weeda, Xie Weifeng, Emmanuel Weiss, Carola Wempe, Ronald Wesselink, Paulina Walczak-Wieteska, Vincent Willems, Xavier Wittebole, Siong Ling Danny Wong, Phunsup Wongsurakiat, Qiu Wusi, Liang Xia, Zhou Li-Xin, Lei Xu, Seyhan Yagar, Saidu Yusuf Yakubu, Masaki Yamasaki, Jing Yan, Congshan Yang, Timothy Yap, Jilu Ye, Ling Ye, Yu Chang Yeh, Aline Camille Yehia, Hüseyin Oguz Yilmaz, Mesut Yilmaz, Chengfen Yin, Yongjie Yin, Pan Yiping, Yerlan Ymbetzhanov, Aimee Yonan, Wang Yongqiang, Hideki Yoshida, Oujidi Younes, Meredith Young, Mikail Yüksel, Jean-Ralph Zahar, Ahmed Zaki, Epaminondas Zakynthinos, Farid Zand, N. Zappela, Rafael Zaragoza, Mohammad Amin Zargar, Amine Ali Zeggwagh, Pinar Zeyneloglu, Jiu Zhi Zhang, Min Zhang, Zhidan Zhang, Tang Zhanhong, Juan Zhao, Peng Zhao, Wang Zhen, Suming Zhou, Biljana Zlojutro, Artemis Zormpa, Nadezhda Zubareva, Francesco Zuccaro, EUROBACT-2 Study Group, Jeffrey Lipman, Alexis Tabah, Hamish Pollock, Ben Margetts, Andrew Udy, Meredith Young, Neeraj Bhadange, Steven Tyler, Anne Ledtischke, Mackenzie Finnis, Jyotsna Dwivedi, Manoj Saxena, Vishwanath Biradar, Natalie Soar, Vineet Sarode, David Brewster, Adrian Regli, Elizabeth Weeda, Samiul Ahmed, Cheryl Fourie, Kevin Laupland, Mahesh Ramanan, James Walsham, Jason Meyer, Edward Litton, Anna Maria Palermo, Timothy Yap, Ege Eroglu, Antony George Attokaran, C’havala Jaramillo, Khalid Mahmood Khan Nafees, Nurhikmahtul Aqilah Haji Abd Rashid, Haji Adi Muhamad Ibnu Walid, Tomas Mon, P. Dhakshina Moorthi, Shah Sudhirchandra, Dhadappa Damodar Sridharan, Qiu Haibo, Xie Jianfeng, Lu Wei-Hua, Wang Zhen, Chuanyun Qian, Jili Luo, Xiaomei Chen, Hao Wang, Peng Zhao, Juan Zhao, Qiu Wusi, Chen Mingmin, Lei Xu, Chengfen Yin, Ruilan Wang, Jinfeng Wang, Yongjie Yin, Min Zhang, Jilu Ye, Chungfang Hu, Suming Zhou, Min Huang, Jing Yan, Yan Wang, Bingyu Qin, Ling Ye, Xie Weifeng, Li Peije, Nan Geng, Lowell Ling, Yoshiro Hayashi, Toshiyuki Karumai, Masaki Yamasaki, Satoru Hashimoto, Koji Hosokawa, Jun Makino, Takeo Matsuyoshi, Akira Kuriyama, Hidenobu Shigemitsu, Yuka Mishima, Michio Nagashima, Hideki Yoshida, Koichiro Omori, Hiroshi Rinka, Hiroki Saito, Kaori Atobe, Hideaki Kato, Shunsuke Takaki, M. Shahnaz Hasan, Muhamad Fadhil Hadi Jamaluddin, Lee See Pheng, Sheshendrasurian Visvalingam, Mun Thing Liew, Siong Ling Danny Wong, Kean Khang Fong, Hamizah Bt Abdul Rahman, Zuraini Md Noor, Lee Kok Tong, Abd. Hamid Azman, Mohd Zulfakar Mazlan, Saedah Ali, Anton Abello, Kyeongman Jeon, Sang-Min Lee, Sunghoon Park, Seung Yong Park, Sung Yoon Lim, Qing Yuan Goh, Shin Yi Ng, Sui An Lie, Andrea Lay Hoon Kwa, Ken Junyang Goh, Andrew Yunkai Li, Caroline Yu Ming Ong, Jia Yan Lim, Jessica Lishan Quah, Kangqi Ng, Louis Xiang Long Ng, Yu Chang Yeh, Nai-Kuan Chou, Cong-Tat Cia, Ting-Yu Hu, Li-Kuo Kuo, Shih-Chi Ku, Phunsup Wongsurakiat, Yutthana Apichatbutr, Supattra Chiewroongroj, Rashid Nadeem, Ashraf El Houfi, Adel Alsisi, Amr Elhadidy, Mina Barsoum, Nermin Osman, Tarek Mostafa, Mohamed Elbahnasawy, Ahmed Saber, Amer Aldhalia, Omar Elmandouh, Ahmed Elsayed, Merihan A. Elbadawy, Ahmed K. Awad, Hanan M. Hemead, Farid Zand, Maryam Ouhadian, Seyed Hamid Borsi, Zahra Mehraban, Davood Kashipazha, Fatemeh Ahmadi, Mohsen Savaie, Farhad Soltani, Mahboobeh Rashidi, Reza Baghbanian, Fatemeh Javaherforoosh, Fereshteh Amiri, Arash Kiani, Mohammad Amin Zargar, Ata Mahmoodpoor, Fatemeh Aalinezhad, Gholamreza Dabiri, Golnar Sabetian, Hakimeh Sarshad, Mansoor Masjedi, Ramin Tajvidi, Abdullah Khudhur Ahmed, Pierre Singer, Ilya Kagan, Merav Rigler, Daniel Belman, Phillip Levin, Belal Harara, Adei Diab, Fayez Abillama, Rebecca Ibrahim, Aya Fares, Ahmad Buimsaedah, Marwa Gamra, Ahmed Aqeelah, Almajdoub Ali Mohammed Ali, Ahmed Gaber Sadik Homaidan, Bushray Almiqlash, Hala Bilkhayr, Ahmad Bouhuwaish, Ahmed Sa Taher, Eman Abdulwahed, Fathi A. Abousnina, Aisha Khaled Hdada, Rania Jobran, Hayat Ben Hasan, Rabab Shaban Ben Hasan, Issam Serghini, Rachid Seddiki, Brahim Boukatta, Nabil Kanjaa, Doumiri Mouhssine, Maazouzi Ahmed Wajdi, Tarek Dendane, Amine Ali Zeggwagh, Brahim Housni, Oujidi Younes, Abdelhamid Hachimi, A. Ghannam, Z. Belkhadir, Sarah Amro, Mustafa Abu Jayyab, Ali Ait Hssain, Abdurahaman Elbuzidi, Edin Karic, Marcus Lance, Shaikh Nissar, Hend Sallam, Omar Elrabi, Ghaleb A. Almekhlafi, Maher Awad, Ahmed Aljabbary, Mohammad Karam Chaaban, Natalia Abu-Sayf, Mazzeh Kiwan, Mohammad Al-Jadaan, Lubna Bakr, Mounir Bouaziz, Olfa Turki, Walid Sellami, Pablo Centeno, José Oscar Acevedo, Patricia Mabel Lopez, Rubén Fernández, Matías Segura, Yanina Nuccetelli, Pablo Montefiore, Luis Felipe Reyes, Silvio A. Ñamendys-Silva, Juan P. Romero-Gonzalez, Mariana Hermosillo, Roberto Alejandro Castillo, Jesús Nicolás Pantoja Leal, Candy Garcia Aguilar, Mara Ocotlan Gonzalez Herrera, Missael Vladimir Espinoza Villafuerte, Manuel Lomeli-Teran, Jose G. Dominguez-Cherit, Adrian Davalos-Alvarez, Luis Sánchez-Hurtado, Brigitte Tejeda-Huezo, Orlando R. Perez-Nieto, Ernesto Deloya Tomas, Guy Francois, Liesbet De Bus, Jan De Waele, Isabelle Hollevoet, Wouter Denys, Marc Bourgeois, Jean-Baptiste Mesland, Pierre Henin, Lionel Haentjens, Patrick Biston, Cindérella Noel, Nathalie Layos, Benoît Misset, Nicolas De Schryver, Nicolas Serck, Xavier Wittebole, Elisabeth De Waele, Godelive Opdenacker, Pedja Kovacevic, Biljana Zlojutro, Aida Custovic, Ina Filipovic-Grcic, Radovan Radonic, Ana Vujaklija Brajkovic, Jasminka Persec, Sanja Sakan, Mario Nikolic, Hrvoje Lasic, Jean-François Timsit, Etienne Ruppe, Stephane Ruckly, Philippe Montravers, Marc Leone, Charlotte Arbelot, Juliette Patrier, Nathalie Zappella, Thierry Dulac, Jérémy Castanera, Johann Auchabie, Anthony Le Meur, A. Marchalot, M. Beuzelin, Alexandre Massri, Charlotte Guesdon, Etienne Escudier, Philippe Mateu, Jérémy Rosman, Olivier Leroy, Serge Alfandari, Alexandru Nica, Bertrand Souweine, Elisabeth Coupez, Thibault Duburcq, Eric Kipnis, Perrine Bortolotti, Mathieu Le Souhaitier, Jean-Paul Mira, Pierre Garcon, Matthieu Duprey, Martial Thyrault, Rémi Paulet, François Philippart, Marc Tran, Cédric Bruel, Emmanuel Weiss, Sylvie Janny, Arnaud Foucrier, Pierre-François Perrigault, Flora Djanikian, François Barbier, Marc Gainnier, Jérémy Bourenne, Guillaume Louis, Roland Smonig, Laurent Argaud, Thomas Baudry, Armand Mekonted Dessap, Keyvan Razazi, Pierre Kalfon, Gaëtan Badre, Romaric Larcher, Jean-Yves Lefrant, Claire Roger, Benjamine Sarton, Stein Silva, Sophie Demeret, Loïc Le Guennec, Shidasp Siami, Christelle Aparicio, Guillaume Voiriot, Muriel Fartoukh, Claire Dahyot-Fizelier, Nadia Imzi, Kada Klouche, Hendrik Bracht, Sandra Hoheisen, Frank Bloos, Daniel Thomas-Rueddel, Sirak Petros, Bastian Pasieka, Simon Dubler, Karsten Schmidt, Antje Gottschalk, Carola Wempe, Philippe Lepper, Carlos Metz, Dmitriy Viderman, Yerlan Ymbetzhanov, Miras Mugazov, Yelena Bazhykayeva, Zhannur Kaligozhin, Baurzhan Babashev, Yevgeniy Merenkov, Talgat Temirov, Kostoula Arvaniti, Dimitrios Smyrniotis, Vasiliki Psallida, Georgios Fildisis, Evangelos Kaimakamis, Cristina Iasonidou, Sofia Papoti, Maria Vasileiou, Vasiliki Romanou, Vasiliki Koutsoukou, Mariana Kristina Matei, Leora Moldovan, Ilias Karaiskos, Harry Paskalis, Kyriaki Marmanidou, M. Papanikolaou, C. Kampolis, Marina Oikonomou, Evangelos Kogkopoulos, Charikleia Nikolaou, Anastasios Sakkalis, Marinos Chatzis, Maria Georgopoulou, Anna Efthymiou, Vasiliki Chantziara, Aikaterini Sakagianni, Zoi Athanasa, Eirini Papageorgiou, Fadi Ali, Georges Dimopoulos, Mariota Panagiota Almiroudi, Polychronis Malliotakis, Diamantina Marouli, Vasiliki Theodorou, Ioannis Retselas, Vasilios Kouroulas, Georgios Papathanakos, Gabriele Sales, Gennaro De Pascale, Luca Maria Montini, Simone Carelli, Joel Vargas, Valentina Di Gravio, Daniele Roberto Giacobbe, Angelo Gratarola, Elisa Porcile, Michele Mirabella, Ivan Daroui, Giovanni Lodi, Francesco Zuccaro, Maria Grazia Schlevenin, Paolo Pelosi, Denise Battaglini, Andrea Cortegiani, Mariachiara Ippolito, Davide Bellina, Andrea Di Guardo, Lorella Pelagalli, Marco Covotta, Monica Rocco, Silvia Fiorelli, Anna Chiara Rizzo, Adam Mikstacki, Barbara Tamowicz, Irmina Kaptur Komorowska, Anna Szczesniak, Jozef Bojko, Anna Kotkowska, Paulina Walczak-Wieteska, Dominika Wasowska, Tomasz Nowakowski, Hanna Broda, Mariusz Peichota, Iwona Pietraszek-Grzywaczewska, Ignacio Martin-Loeches, Alessandra Bisanti, Pedro Póvoa, Nuno Cartoze, Tiago Pereira, Madalena Alves, Ana Josefina Pinheiro Marques, Ana Rios Pinto, Andriy Krystopchuk, Ana Teresa, Jose De Almeida, António Manuel Pereira de Figueiredo, Isabel Botelho, Tiago Duarte, Vasco Costa, Rui Pedro Cunha, Elena Molinos, Tito da Costa, Sara Ledo, Joana Queiró, Dulce Pascoalinho, Cristina Nunes, José Pedro Moura, Énio Pereira, António Carvalho Mendes, C. C. Iliescu, Liana Valeanu, Serban Bubenek-Turconi, Ioana Marina Grintescu, Cristian Cobilinschi, Daniela Carmen Filipescu, Cornelia Elena Predoi, Dana Tomescu, Mihai Popescu, Alexandra Marcu, Ioana Grigoras, Olguta Lungu, Alexey Gritsan, Anastasia Anderzhanova, Yulia Meleshkina, Marat Magomedov, Nadezhda Zubareva, Maksim Tribulev, Denis Gaigolnik, Natala Vistovskaya, Maria Chukina, Vladislav Belskiy, Mikhail Furman, Ricard Ferrer Rocca, Maria Martinez, Vanessa Casares, Ricard Mellado Artigas, Paula Vera, Matias Flores, Joaquin Amador Amerigo, Maria Pilar Gracia Arnillas, Rosana Munoz Bermudez, Fernando Armestar, Beatriz Catalan, Regina Roig, Laura Raguer, María Dolores Quesada, Emilio Diaz Santos, Gemma Gomà, Alejandro Ubeda, Maria Salgado, Lorena Forcelledo Espina, Emilio Garcia Prieto, M. Rodriguez, Emilio Maseda, Alejandro Suarez De La Rica, J. Ignacio Ayestaran, Mariana Novo, Miguel Angel Blasco-Navalpotro, Alberto Orejas Gallego, Fredrik Sjövall, Dzana Spahic, Carl Johan Svensson, Michael Haney, Alicia Edin, Joyce Åkerlund, Lina De Geer, Niccolò Buetti, Josef Prazak, Jl Pagani, S. Abed-Maillard, Murat Akova, Abdullah Tarik Aslan, Arif Timuroglu, Sesin Kocagoz, Hulya Kusoglu, Selcuk Mehtap, Solakoglu Ceyhun, Neriman Defne Altintas, Leyla Talan, Bircan Kayaaslan, Ayse Kaya Kalem, Ibrahim Kurt, Murat Telli, Barcin Ozturk, Çigdem Erol, Emine Kubra Dindar Demiray, Sait Çolak, Türkay Akbas, Kursat Gundogan, Ali Sari, Canan Agalar, Onur Çolak, Mesut Yilmaz, Burcu Tunay, Rumeysa Cakmak, Nese Saltoglu, Ridvan Karaali, Iftihar Koksal, Firdevs Aksoy, Kemal Tolga Saracoglu, Yeliz Bilir, Seda Guzeldag, Gulden Ersoz, Guliz Evik, Hulya Sungurtekin, Cansu Ozgen, Cem Erdogan, Yunus Gürbüz, Nilgün Altin, Yasar Bayindir, Yasemin Ersoy, Senay Goksu, Ahmet Akyol, Ayse Batirel, Sabahat Cagan Aktas, Andrew Conway Morris, Matthew Routledge, Ari Ercole, David Antcliffe, Roceld Rojo, Kate Tizard, Maria Faulkner, Amanda Cowton, Melanie Kent, Ashok Raj, Artemis Zormpa, George Tinaslanidis, Reena Khade, Tomasz Torlinski, Randeep Mulhi, Shraddha Goyal, Manan Bajaj, Marina Soltan, Aimee Yonan, Rachael Dolan, Aimee Johnson, Caroline Macfie, James Lennard, Maie Templeton, Sonia Sousa Arias, Uwe Franke, Keith Hugill, Hollie Angell, Benjamin J. Parcell, Katherine Cobb, Stephen Cole, Tim Smith, Clive Graham, Jaroslav Cerman, Allison Keegan, Jenny Ritzema, Amanda Sanderson, Ashraf Roshdy, Tamas Szakmany, Tom Baumer, Rebecca Longbottom, Daniel Hall, Kate Tatham, S. Loftus, A. Husain, E. Black, S. Jhanji, Rao Baikady, Peter Mcguigan, Rachel Mckee, Santhana Kannan, Supriya Antrolikar, Nicholas Marsden, Valentina Della Torre, Dorota Banach, Ahmed Zaki, Matthew Jackson, Moses Chikungwa, Ben Attwood, Jamie Patel, Rebecca E. Tilley, Sally K. Humphreys, Paul Jean Renaud, Anton Sokhan, Yaroslava Burma, Wendy Sligl, Nadia Baig, Lorena McCoshen, Demetrios J. Kutsogiannis, Patricia Thompson, Tayne Hewer, Raihan Rabbani, Shihan Mahmud Redwanul Huq, Rajib Hasan, Mohammad Motiul Islam, Mohan Gurjar, Arvind Baronia, Nikhil Kothari, Ankur Sharma, Saurabh Karmakar, Priya Sharma, Janardan Nimbolkar, Pratit Samdani, Vaidyanathan R, Noor Ahmedi Rubina, Nikhilesh Jain, Madhumati Pahuja, Ritu Singh, Syed Nabeel Muzaffar, Ahmad Ozair, Suhail Sarwar Siddiqui, Payel Bose, Avijatri Datta, Darshana Rathod, Mayur Patel, M. K. Renuka, Sailaja K. Baby, Carol Dsilva, Jagadish Chandran, Pralay Ghosh, Sudipta Mukherjee, Kaladhar Sheshala, Krushna Chandra Misra, Saidu Yusuf Yakubu, Euphemia Mgbosoro Ugwu, Ibironke Desalu, Gabriel Asiyanbi, Motunrayo Oladimeji, Olusola Idowu, Fowotade Adeola, Mervyn Mer, Melanie Cree, Ali Adil Ali Karar, Elfayadh Saidahmed, Hytham K. S. Hamid, Shigeki Fujitani, Helmi Sulaiman, Muhammed Elhadi, Khalid Abidi, Gabriela Vidal, Mario Arias, Vasiliki Soulountsi, Matteo Bassetti, Giorgia Montrucchio, José-Artur Paiva, Nádia Guimarães, Oyebola O Adekola, Bashir El Sanousi, Seyed Mohammad Nasirodin Tabatabaei, Natalia Morvillo, Marta Aparicio, Irene Alonzo, Sofie F. M. Vanderhaeghen, Foteini Renta, Antonella Cotoia, Aleksandr Eremenko, M. J. Asensio, Stephan Jakob, Jean-Luc Pagani, Nurcan N. Baykam, Ozlem O. Akdogan, Ahmet Eroglu, Saurav Shekhar, John O. Olatosi, Aaron Mark Hernandez, Fayez Abilama, Ali Aithssain, Arzu Topeli Iskit, ESICM, ESCMID ESGCIP, OUTCOMEREA Network

**Affiliations:** 1 Division of Intensive Care Medicine, Department of Acute Care Medicine, Geneva University Hospitals, Geneva, Switzerland; 2 Faculty of Medicine, University of Geneva, Geneva, Switzerland; 3 School of Medicine, Faculty of Health, Queensland University of Technology, Brisbane, Australia; 4 Intensive Care Unit, Redcliffe Hospital, Redcliffe, Australia; 5 Faculty of Medicine, Mayne Academy of Critical Care, University of Queensland, St Lucia, Australia; 6 Infectious Diseases Clinic, IRCCS Azienda Ospedaliera Metropolitana, Policlinico San Martino, Italy; 7 Department of Health Science, University of Genoa, Genoa, Italya Italy; 8 Department of Intensive Care Services, Royal Brisbane and Women’s Hospital, Brisbane, QLD Australia; 9 School of Medicine, Queensland University of Technology (QUT), Brisbane, QLD Australia; 10 Intensive Care Medicine Service, Centro Hospitalar Universitário de São João, Faculdade de Medicina, Universidade do Porto, Porto, Portugal; 11 Biometry Department, ICUREsearch, Fontaine, France; 12 Medical ICU, La Source Hospital, CHR Orléans, Orléans, France; 13 Research Center for Respiratory Diseases, INSERM, U1100, Tours, 37032 France; 14 INSERM, IAME, U1137, Team DeSCID, Paris, France; 15 APHP Bichat University Hospital Medical and Infectious Diseases ICU Paris, Paris, France; 16 Infection Control Program, Faculty of Medicine, Geneva University Hospitals, World Health Organization Collaborating Centre, Geneva, Switzerland

**Keywords:** Bloodstream infections, Critical care, Fungal infection

## Abstract

**Background:**

The optimal timing of empiric antifungal therapy in patients with suspected candidemia remains uncertain. In practice, many antifungal treatments initiated empirically are eventually found to be unnecessary, exposing patients to potential toxicity, selection of non-*albicans Candida* species, emergence of resistance, and additional costs. The objective of the study was to evaluate whether adequate empiric antifungal therapy initiated within 2 days after blood culture draw was associated with 28-day mortality.

**Methods:**

We performed a pooled secondary analysis of two prospective worldwide observational cohorts (EUROBACT-1 and EUROBACT-2) including adult patients with *Candida* bloodstream infection. The exposure was defined as receipt of adequate antifungal therapy within 2 days after blood culture draw. Propensity scores for receiving early adequate antifungal therapy were estimated using prespecified covariates, and inverse probability of treatment weights were applied in a weighted Cox proportional hazards model to assess the association with 28-day mortality. Prespecified subgroup analyses were conducted according to study cohort, Candida species, and presence of hypotension at the time of bloodstream infection. As a sensitivity analysis, a frailty Cox proportional hazards model including a random center effect was performed.

**Results:**

The final study population included 272 patients with candidemia from 158 centers, including 81 from EUROBACT-1 and 191 from EUROBACT-2. One hundred and ten patients received early adequate antifungal therapy and 162 did not. Overall, 38.6% of patients (105/272) died within 28 days, including 40.9% (45/110) in the early adequate therapy group and 37.0% (60/162) in the non-early adequate therapy group. In the weighted Cox model, early adequate antifungal therapy was not significantly associated with 28-day mortality (HR 1.23, 95% CI 0.82–1.83, *p* = 0.314). Results were consistent across subgroup analyses according to study cohort, *Candida* species, the presence of hypotension at the time of bloodstream infection, as well as in the frailty Cox sensitivity analysis.

**Conclusions:**

Early adequate antifungal therapy was not independently associated with improved 28-day survival in ICU patients with candidemia. These findings support a more targeted approach to early empirical antifungal treatment and underscore the need to identify patient subgroups most likely to benefit from immediate therapy.

**Supplementary Information:**

The online version contains supplementary material available at https://doi.org/10.1186/s13054-026-06373-3.

## Background

Bloodstream infections (BSIs) are common in intensive care unit (ICU) patients and are associated with substantial morbidity and mortality with mortality rates reaching up to 40% in a recent European cohort [1]. It is associated with prolonged hospital stay and major healthcare costs [1–4]. Among ICU-acquired BSIs, fungal BSIs represent an important subgroup with candidemia being the predominant fungal infection [2].

Several risk factors for candidemia have been identified in the past literature, including broad-spectrum antibiotic exposure, Candida colonization, central venous catheter use, total parenteral nutrition, blood transfusion, and several ICU-related invasive procedures [5]. Prognostic factors for mortality in this population have been reported, such as patient severity on admission, need for mechanical ventilation, and ICU admission for a medical reason [2, 6]. However, the association between delayed appropriate antifungal therapy and mortality remains controversial. Antifungal treatment decision-making in suspected candidemia carries on the one hand the risk of delayed appropriate treatment and on the other hand the risk of unnecessary empirical exposure, which is associated with potential toxicity, emergence of resistance, selection of non-albicans species and costs [7–10]. Some studies have reported improved survival with earlier active antifungal treatment [7, 11–13], whereas others did not find an association between a treatment delay and mortality [2, 6, 14–16]. Across studies, early appropriate therapy has generally been defined as the administration, within the first 24–48 h after blood culture draw, of an antifungal agent active against the isolated *Candida* species and given at an adequate dose.

Unfortunately, many of these studies were limited by relatively small sample sizes or by restricted geographic representation or older data that may not reflect current practice, which may limit generalizability across countries and clinical practices [7, 11, 17, 18]. Current guidelines recommend rapid initiation of antifungal therapy when fungal infection is suspected, favor echinocandins as first-line treatment, and emphasize the importance of source control, particularly catheter removal [19–21]. Conventional blood cultures, species identification, and susceptibility testing frequently require at least 48 h [22]. It remains unclear whether empirical antifungal therapy should be initiated at the time blood cultures are obtained in ICU patients with suspected infection before the causative pathogen is known, or whether antifungal treatment can safely be deferred until microbiological confirmation of candidemia without adversely affecting patient outcomes.

In the present study, we pooled data from the EUROBACT-1 [23] and EUROBACT-2 [24] studies to assess whether early appropriate antifungal therapy, defined as an adequate treatment administered within the first 2 days following blood culture draw, is associated with mortality.

## Methods

### Setting

The EUROBACT studies were prospective observational international cohort studies focused on hospital-acquired bloodstream infections (HABSI), with EUROBACT-1 being conducted between 2010 and 2011 and EUROBACT-2 between 2019–2021 [23, 24]. The detailed methodologies of both studies have been previously described [23, 24].

Endorsement, logistic, and financial support was obtained from the European Society of Clinical Microbiology and Infectious Diseases (ESCMID) study Group for Infections in Critically Ill Patients (ESGCIP) and the European Society of Intensive Care Medicine (ESICM). An operational committee (AT, JFT, FB, SR, and NB) oversaw all study operations. National coordinators (NCs) recruited participating centres and applied for regulatory and ethical approvals.

### Centers selection

The included centres were ICUs, defined as a unit designed to manage patients with organ failure and able to provide invasive mechanical ventilation for at least 24 h.

For EUROBACT-1, a total of 162 ICUs in 24 countries participated in the study. The international database was declared to the CNIL (Commission Nationale de l’Informatique et des Libertés). The study was conducted according to local and national regulations for each participating ICU.

For EUROBACT-2, a total of 333 centers in 52 countries participated in the study (NCT03937245, registered 3 May 2019). The study was approved by the ethics Committee of the Royal Brisbane & Women’s Hospital Human Research (LNR/2019/QRBW/48376). Each study site then obtained ethical and governance approvals according to the local regulations in place [24].

The two studies were reported in accordance with the STrengthening the Reporting of OBservational studies in Epidemiology (STROBE) guidelines [25].

### Patient selection

Adult patients (≥ 18 years old) with a first episode of HABSI treated in ICU were enrolled. HABSI was defined as a positive blood culture collected at least 48 h after hospital admission. Patients were eligible if the blood culture was collected in the ICU or if they were transferred to the ICU for management of HABSI. Only the first episode of HABSI was considered, with only one HABSI episode included per patient.

For the present analysis, we retrieved individual patient-level data for all patients with microbiologically documented *Candida* BSI. To limit immortal time bias, we used a 48-hour landmark time-point after blood culture sampling, excluding patients who died before this time point because they could not have been adequately exposed to early antifungal therapy. We also excluded breakthrough *Candida* BSI, defined as *Candida* growth in blood cultures during ongoing antifungal treatment, but not antifungal prophylaxis, as these episodes may represent a distinct and more difficult-to-treat clinical entity.

### Data collection and definition of the exposure

A center-level form was completed to describe ICU characteristics and organization. It included the type of ICU, hospital funding, the number of nurses and physicians per patient, the availability of an infectious diseases clinician, and local antimicrobial stewardship and pharmacological support, for which aminoglycoside therapeutic drug monitoring (TDM) availability was used as a surrogate [26]. For each patient, demographic data, comorbidities (Charlson comorbidity index [27]), immunosuppression (defined as corticosteroid therapy, chemotherapy, AIDS, or hematologic malignancy, including lymphoma or leukemia [28]), ICU admission diagnosis (medical vs. surgical), clinical characteristics at ICU admission, and severity scores (SAPS II) were prospectively collected. On the HABSI day, defined as the day the blood culture was collected, clinical and laboratory variables, SOFA score, presumed source of infection, and source control data were recorded. Sources of HABSI were recorded based on clinical likelihood according to the treating clinician. Primary HABSI was defined as no clear portal of entry or source of infection. The organisms causing HABSI and their antimicrobial susceptibility test results were recorded according to local policies.

Early adequate antifungal treatment was defined as receipt, within 2 days after blood culture draw, of an antifungal agent with in vitro activity against the isolated *Candida* species and administered at an appropriate dose, defined as fluconazole 800 mg loading dose followed by at least 400 mg daily; caspofungin 70 mg loading dose (100 mg for body weight > 80 kg) followed by 50 mg/day (70 mg/day for body weight > 80 kg); micafungin 100 mg/day; anidulafungin 200 mg loading dose followed by 100 mg/day; or liposomal amphotericin B ≥ 3 mg/kg/day [7, 12]. Treatment timing was based on the recorded treatment date. Prophylactic antifungal use was not considered therapeutic treatment and was distinguished based on the agent, dose, dosing interval, and timing. Appropriateness of antifungal therapy was evaluated for each case by two experts in the field (NB, HW).

Patients were followed until hospital discharge or day 28, whichever occurred first. The primary outcome was 28-day in-hospital mortality.

Data were collected locally by investigators in each participating ICU, and data quality was supported through electronic checks and review of case records for consistency and completeness.

### Statistical analyses

Baseline characteristics were described according to exposure to early adequate antifungal therapy within 2 days after blood culture draw. Continuous variables were expressed as median (IQR) and categorical variables as n (%). Comparisons between groups were performed using the chi-square test or Fisher’s exact test for categorical variables, and the Wilcoxon rank-sum test for continuous variables.

The association between early adequate antifungal therapy and mortality was primarily assessed using a Cox proportional hazards model weighted with stabilized inverse probability of treatment weights derived from the propensity score. The propensity score for receiving early adequate antifungal therapy was estimated using prespecified covariates selected as potential confounders of the association between early adequate antifungal therapy and mortality, including source of infection, source control management, availability of local antimicrobial stewardship and pharmacological support (based on the presence of available aminoglycoside TDM), reason for admission (medical/surgical), need for mechanical ventilation at the time of BSI, SOFA score at the time of BSI, and Charlson comorbidity index [2, 20, 24, 29]. Covariate selection was informed by a directed acyclic graph (DAG) presented in Supplementary Fig. 1. Stabilized inverse probability of treatment weights were then derived from the estimated propensity score and applied to create a weighted pseudopopulation balanced on prespecified measured confounders. Covariate balance before and after weighting was assessed using standardized mean differences and visualized using a Love plot. The proportional hazards assumption was evaluated using Schoenfeld residuals and by graphical inspection of log-log survival plots. Subgroup analyses were performed according to study cohort (EUROBACT-1 vs. EUROBACT-2), presence of hypotension at the time of BSI, *Candida* species, including *C. albicans* versus other *Candida* species and *C. glabrata* versus other *Candida* species, and after restriction to patients with time to positivity ≥ 2 days. This latter analysis was performed to account for potential bias from early microbiological information influencing empirical antifungal adequacy. As a sensitivity analysis, frailty Cox proportional hazards models with center included as a random effect were performed to account for potential center effects across participating sites. Univariable analyses were first performed, followed by a multivariable model adjusted for the same prespecified covariates as in the primary analysis. As an additional sensitivity analysis, we reassessed the association between adequate antifungal therapy and 28-day mortality using a more restrictive exposure definition of treatment initiated within 1 day of blood culture collection, with univariable and multivariable Cox proportional hazards models.

Two-tailed *p*-values ≤ 0.05 were considered statistically significant. All statistical analyses were conducted using Stata version 16.1 (Stata Corp., College Station, TX, USA, 2007) and R (Version 3.5.3).

## Results

### Study population

**Fig. 1:**
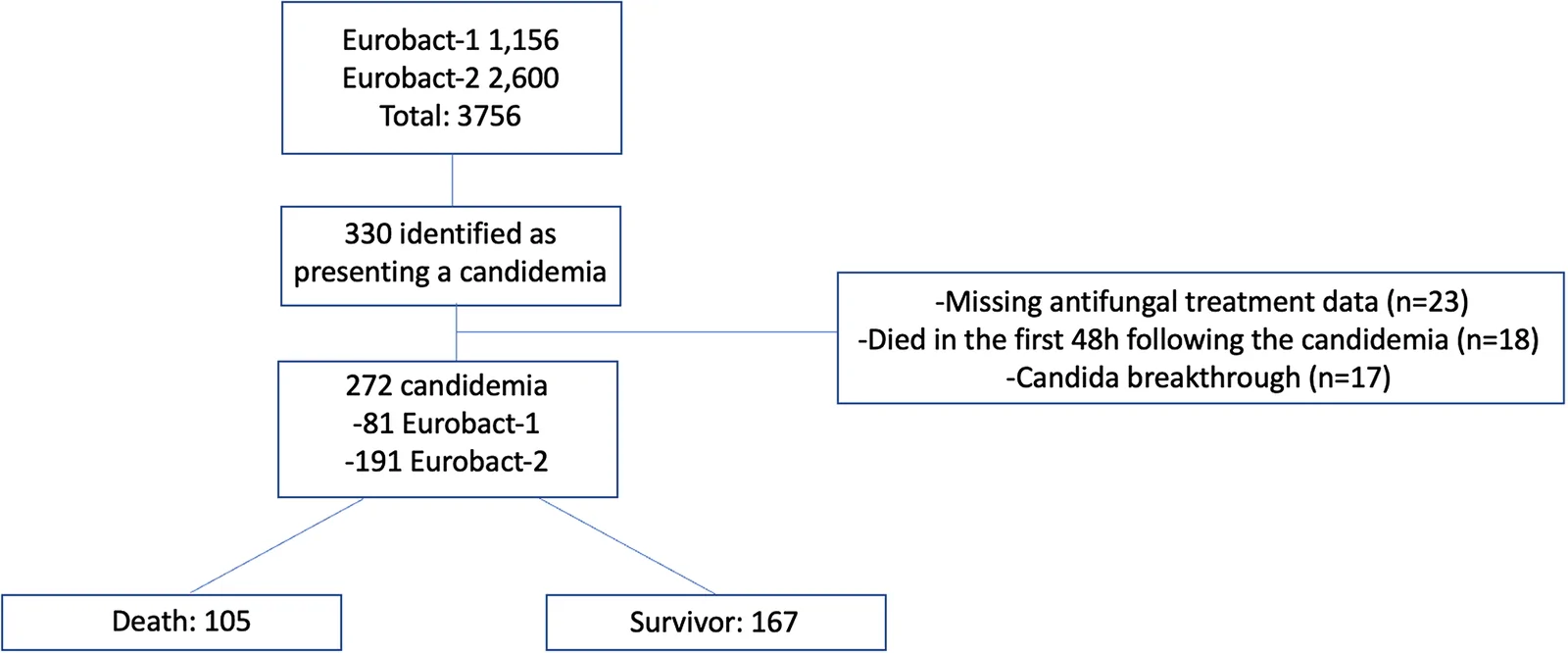
Flowchart of cohort selection in ICU patients with hospital-acquired bloodstream infection. Legend: Flowchart of cohort selection in ICU patients with hospital-acquired bloodstream infection. Of 3,756 patients included in EUROBACT-1 and EUROBACT-2, 330 patients with candidemia were identified. After exclusion of patients with missing antifungal treatment data, death within 2 days of candidemia onset, or breakthrough candidemia, 272 patients were included in the final analysis

**Fig. 2:**
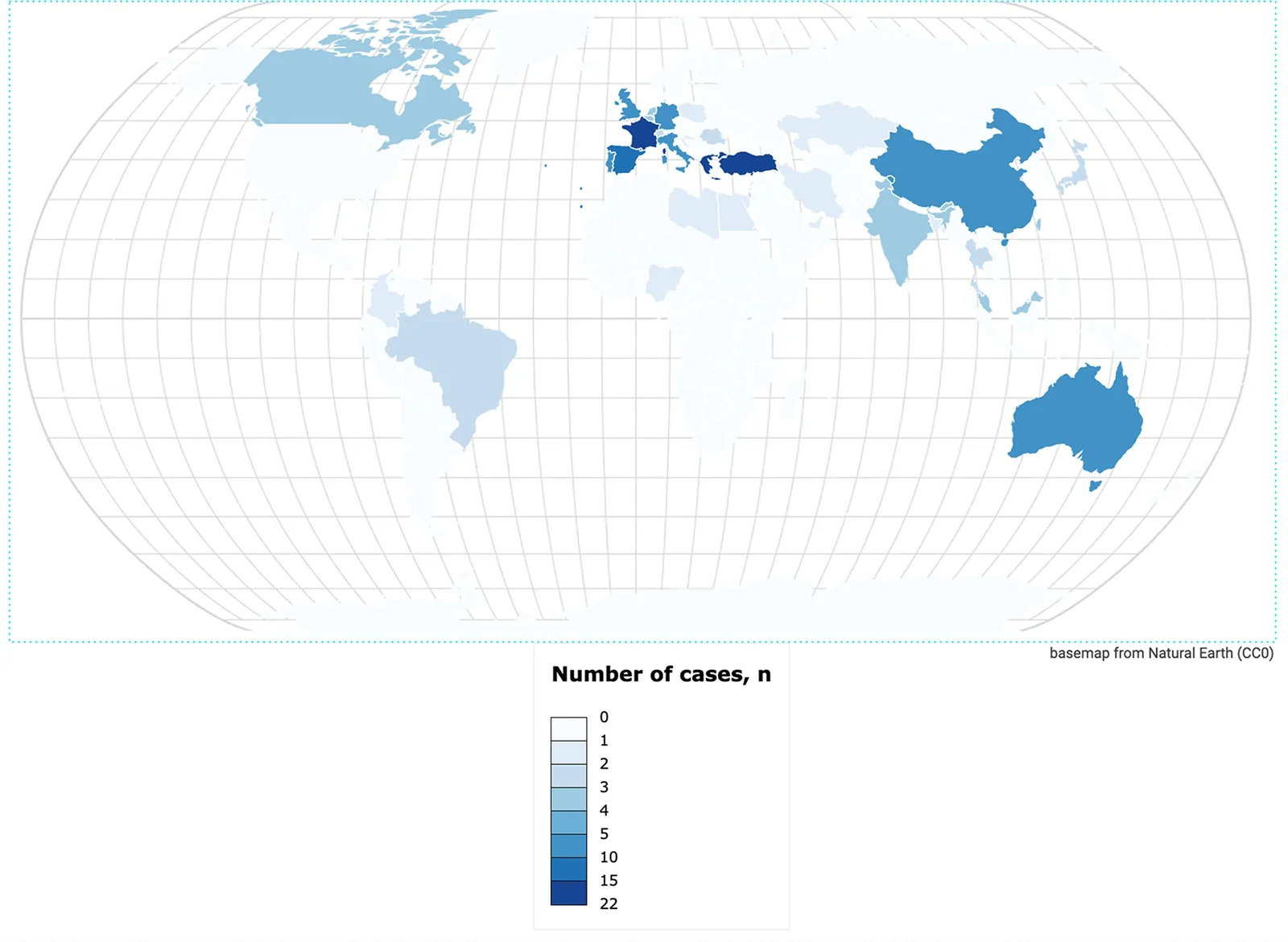
Geographical distribution of the cases included. Legend: Global distribution of included cases by country. The map shows the absolute number of patients included in each participating country; darker shading indicates a higher case count. Countries with no included cases are shown in the lightest shade

Among the 3756 patients included in EUROBACT-1 and EUROBACT-2, 330 were identified as having candidemia (Fig. 1). Of these, 23 patients were excluded because of missing antifungal treatment data, 18 because they died within the first 2 days following blood culture draw, and 17 because they had breakthrough candidemia. Details of the 18 patients excluded because of death within 2 days are provided in Supplementary Table 1. The final study population included 272 patients with candidemia from 158 centers: 81 from EUROBACT-1 and 191 from EUROBACT-2, with 110 patients having received early adequate antifungal therapy and 162 not. Among the 162 patients who did not receive adequate antifungal therapy within the first 2 days, 143 (88.3%) eventually received antifungal therapy, whereas 19 (11.7%) never received antifungal therapy during the available follow-up. Geographical distribution of the cases is presented in Fig. 2. Details of the included centers are presented in Supplementary Table 2.

### Centers and patients’ characteristics

Patients were mainly treated in closed ICUs (76.8%, 209/272) and in mixed medical-surgical ICUs (76.5%, 208/272) (Table 1). ICU structure and ICU type did not differ significantly according to exposure to early adequate antifungal therapy. Most patients were treated in centers with an infectious diseases’ consultant available (94.3%, 248/263).

**Table 1:** Center characteristics

Variable	All patients (*n* = 272)	Not adequate (*n* = 162)	Early adequate (*n* = 110)	*p*-value
**Center characteristics**				
ICU structure, closed ICU, n (%)	209 (76.8%)	121 (74.7%)	88 (80.0%)	0.308
ICU type - Medical - Surgical - Mixed	46 (16.9%) 18 (6.6%) 208 (76.5%)	27 (16.7%) 11 (6.8%) 124 (76.5%)	19 (17.3%) 7 (6.4%) 84 (76.4%)	0.984
Number of nurses, median (IQR)	14 (5–32)	12 (5–30)	16 (6–37)	0.078
Number of physicians, median (IQR)	7 (4–10)	6 (4–10)	7 (5–10)	0.152
Infectious disease specialist available, n (%)	248 (94.3%)	149 (94.9%)	99 (93.4%)	0.605
Antibiotic stewardship and pharmacological support available^1^, n (%)	174 (64.0%)	97 (59.9%)	77 (70.0%)	0.088

^1^Defined based on the availability of aminoglycoside therapeutic drug monitoring (TDM)

Patients’ characteristics according to exposure to early adequate antifungal therapy are presented in Table 2. In the overall population, median age was 64.5 years [IQR 52.5–75.5] and 37.1% (101/272) were women. Median Charlson comorbidity index was 2 [IQR 0–3], median SAPS II at ICU admission was 50 [IQR 38.5–64.5], and median SOFA score at the time of BSI was 8 [IQR 6–12].

**Table 2:** Patients’ characteristics by empiric antifungal therapy adequacy within 2 days

Variable	All patients (*n* = 272)	Not adequate (*n* = 162)	Early adequate (*n* = 110)	*p*-value
**Baseline characteristics**				
Sex, female, n (%)	101 (37.1%)	61 (37.7%)	40 (36.4%)	0.829
Age (years), median [IQR]	64.5 [52.5–75.5]	65 [53–77]	64 [51–74]	0.455
BMI (kg/m²), median [IQR]	26.0 [22.0–29.4]	25.4 [22.0–29.4]	26.1 [22.0–29.5]	0.898
Charlson Comorbidity Index, median [IQR]	2 [0–3]	2 [1–3]	2 [0–3]	0.266
Immunosuppression, n (%)	39 (14.3%)	23 (14.2%)	16 (14.5%)	0.936
Ad mission type, n (%)				
-medical -surgical	162 (59.6%) 110 (40.4%)	115 (71.0%) 47 (20.0%)	75 (68.2%) 35 (31.8%)	0.621
SAPS II at ICU admission, median [IQR]	50 [38.5–64.5]	50 [38–65]	50 [39–63]	0.858
**Sepsis features on the day of BSI**				
SOFA score, median [IQR]	8 [6–12]	8 [5–12]	9 [7–13]	0.014
Maximum temperature (°C), median [IQR]	37.3 [36.7–38.2]	37.3 [36.7–38.2]	37.3 [36.6–38.4]	0.701
Glasgow Coma Scale, median [IQR]	13 [7–15]	13 [7–15]	13 [8–15]	0.765
Sepsis-associated AKI, n (%)	150/271 (55.4%)	87/162 (53.7%)	63/109 (57.8%)	0.506
Fever, n (%)	163 (59.9%)	89 (54.9%)	74 (67.3%)	0.042
Hypotension, n (%)	108/254 (42.5%)	55/152 (36.2%)	53/102 (52.0%)	0.013
**Organ support during ICU stay and length of stay**				
Mechanical ventilation, n (%)	236/271 (87.1%)	142/161 (88.2%)	94/110 (85.5%)	0.508
Renal replacement therapy, n (%)	106 (39.0%)	56 (34.6%)	50 (45.5%)	0.071
ICU length of stay in days, median [IQR]	23 [14–35]	23 [15–36]	20 [12–34]	0.092
**Source of infection**				
Source of infection, n (%)				
- Catheter - Abdominal - Other	127 (46.7%) 43 (15.8%) 102 (37.5%)	79 (48.8%) 23 (14.2%) 60 (37.0%)	48 (43.6%) 20 (18.2%) 42 (38.2%)	0.592
Source control, n (%)				
-not needed -required and complete -required but not achieved	106 (39.0%) 136 (50.0%) 30 (11.0%)	61 (37.7%) 78 (48.1%) 23 (14.2%)	45 (40.9%) 58 (52.7%) 7 (6.4%)	0.129
**Candida species and antifungal treatment in the first 2 days**				
Blood culture obtained in the ICU, n(%)	256 (94.1%)	156 (96.3%)	100 (90.9%)	0.072
Candida species, n (%)				
- *C. albicans* *- C. glabrata* *- C. parapsilosis* *- C. tropicalis* - Other *Candida*	128 (47.1%) 47 (17.3%) 50 (18.4%) 22 (8.1%) 25 (9.2%)	72 (44.4%) 32 (19.8%) 30 (18.5%) 9 (5.6%) 19 (11.7%)	56 (50.9%) 15 (13.6%) 20 (18.2%) 13 (11.8%) 6 (5.5%)	0.092
Antifungal treatment class, n (%)				
- Echinocandin - Non-echinocandin antifungal - No antifungal in the first 2d	74 (27.2%) 50 (18.4%) 148 (54.4%)	2 (1.2%) 12 (7.4%) 148 (91.4%)	72 (65.5%) 38 (34.5%) 0 (0.0%)	< 0.001
**Outcome**				
28-day mortality, n (%)	105 (38.6%)	60 (37.0%)	45 (40.9%)	0.520

BMI, body mass index; AKI, acute kidney injury, with diagnosis based on clinician assessment

Baseline demographic characteristics, comorbidities, and severity at ICU admission were overall similar between patients receiving early adequate therapy and those who did not. Patients who received early adequate therapy had a higher SOFA score at the time of BSI (9 [IQR 7–13] vs. 8 [IQR 5–12], *p* = 0.014). At the time of BSI, fever and hypotension were more frequently observed among patients who received early adequate antifungal therapy than among those who did not (fever: 67.3% (74/110) vs. 54.9% (89/162), *p* = 0.042; hypotension: 52.0% (53/102) vs. 36.2% (55/152), *p* = 0.013). Overall, 38.6% (105/272) of patients died within 28 days, including 40.9% (45/110) in the early adequate therapy group and 37.0% (60/162) in the non-early adequate therapy group (*p* = 0.520).

### Candida species, source of infection, source control and antifungal treatment

The most frequent species was *Candida albicans* (47.1%, 128/272), followed by *Candida parapsilosis* (18.4%, 50/272). Species distribution did not differ significantly according to adequacy of early treatment (*p* = 0.092).

Central line associated BSI was the most frequent presumed source of candidemia (46.7%, 127/272). The distribution of infection source was similar across groups. Source control was required and not achieved in 14.2% (23/162) of patients who did not receive early adequate therapy and 6.4% (7/110) of those who did, with no statistically significant difference between groups (*p* = 0.129).

Regarding antifungal treatment received within the first 2 days, echinocandins were the most frequent agents among patients receiving early adequate therapy (65.5% (72/110)). In the non-adequate group, 91.4% (148/162) did not receive any antifungal treatment within the first 2 days, 1.2% (2/162) received an echinocandin, and 7.4% (12/162) received a non-echinocandin antifungal agent (*p* < 0.001).

Details on the time to adequate antifungal therapy are shown in Supplementary Fig. 2. Overall, 66 patients (24.3%) received adequate antifungal therapy within 1 day, 44 (16.2%) between 1 and 2 days, 72 (26.5%) between > 2 and ≤ 5 days, and 90 (33.1%) after > 5 days or never.

### IPTW-weighted Cox analysis

**Fig. 3:**
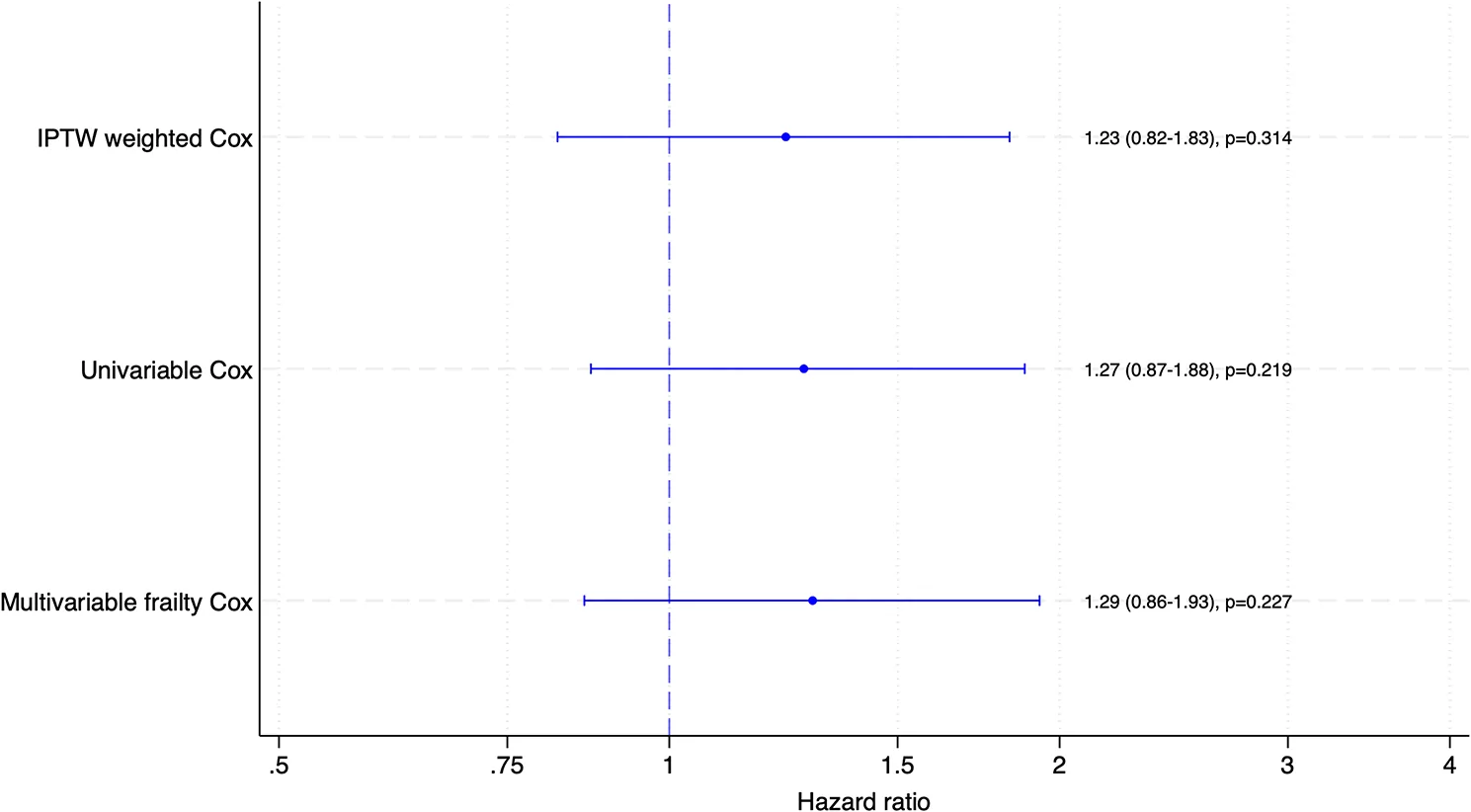
Forest plot showing the association between early adequate antifungal therapy and 28-day mortality across Cox regression models. Legend: Association between early adequate antifungal therapy and 28-day mortality. Hazard ratios (HRs) with 95% confidence intervals are shown for the IPTW-weighted Cox model, univariable Cox model, and multivariable frailty Cox model including a center-level random effect. The dashed vertical line indicates HR = 1. IPTW, inverse probability of treatment weighting

Inverse probability of treatment weighting analysis based on a propensity score was performed. Covariate balance before and after weighting was assessed using standardized mean differences and is shown in Supplementary Fig. 3. In the weighted Cox model, early adequate antifungal therapy was not associated with 28-day mortality (HR 1.23, 95% CI 0.82–1.83; *p* = 0.314) (Fig. 3).

### Frailty Cox model

Supplementary Fig. 4 shows the Kaplan–Meier survival curves for 28-day mortality according to receipt of early adequate antifungal therapy. Using univariable frailty Cox regression, early adequate antifungal therapy was not significantly associated with 28-day mortality (HR 1.27, 95% CI 0.87–1.88; *p* = 0.219). Using a multivariable frailty Cox model adjusted for prespecified confounders and clinically relevant risk factors, the association remained non-significant (HR 1.29, 95% CI 0.86–1.93; *p* = 0.227, Fig. 3 and Supplementary Table 3). Among covariates included in the multivariable model, the adjustment variable SOFA score at the time of BSI was associated with 28-day mortality (HR per 1-point increase 1.13, 95% CI 1.07–1.18; *p* < 0.001). Source control required but not achieved was associated with higher 28-day mortality compared with source control not needed (HR 2.11, 95% CI 1.09–4.09; *p* = 0.026).

### Subgroup and sensitivity analyses

In prespecified subgroup analyses based on the IPTW-weighted Cox model, results were consistent with the main analysis (Supplementary Table 4). Early adequate antifungal therapy within 2 days was not significantly associated with 28-day mortality in either the EUROBACT-1 cohort (HR 1.26, 95% CI 0.57–2.79; *p* = 0.564) or the EUROBACT-2 cohort (HR 1.25, 95% CI 0.78–1.99; *p* = 0.347). Similarly, no significant association was observed in patients with *Candida albicans* BSI (HR 1.42, 95% CI 0.79–2.54; *p* = 0.243) or other Candida species BSI (HR 1.07, 95% CI 0.62–1.87; *p* = 0.799). In additional species-specific analyses, no significant association was observed in patients with *Candida glabrata* BSI (HR 0.44, 95% CI 0.15–1.29; *p* = 0.137) or in patients with other *Candida species* than *C. glabrata* (HR 1.44, 95% CI 0.93–2.22; *p* = 0.103). Stratification by the presence of hypotension at the time of BSI also presented similar findings, with no significant association in patients with hypotension (HR 1.58, 95% CI 0.89–2.81; *p* = 0.116) and in those without hypotension (HR 1.03, 95% CI 0.57–1.85; *p* = 0.923). In an additional sensitivity analysis restricted to patients with available time-to-positivity data and excluding those with time to positivity < 2 days, the association between early adequate antifungal therapy and 28-day mortality remained non-significant (HR 1.09, 95% CI 0.62–1.90; *p* = 0.771).

In an additional sensitivity analysis using a 1-day treatment window, 66/272 (24.3%) patients met this definition, with similar results and no significant association with 28-day mortality (Supplementary Table 5).

## Discussion

In this secondary analysis of the prospective observational EUROBACT-1 and EUROBACT-2 cohorts, which included 272 patients with candidemia from 158 centers worldwide, mortality was high (39%), and no association was found between empiric adequate antifungal treatment administered within the first 2 days after blood culture draw and 28-day mortality. Failure to achieve source control when needed was associated with poor outcome. Findings were consistent across prespecified subgroup analyses according to *Candida* species, presence of hypotension at the time of BSI, and study cohort, as well as in the sensitivity frailty Cox analysis.

Mortality from candidemia is high, and early antifungal treatment is recommended when candidemia is suspected, particularly when clinical suspicion is high. However, the survival benefit of empiric antifungal treatment in patients without definite candidemia at treatment initiation remains uncertain. Moreover, early adequate antifungal therapy within 2 days remains uncommon in fungal BSI [11]. Several earlier studies, mostly performed outside the ICU, suggested a survival benefit from early antifungal therapy. Morrell et al. and Garey et al. both reported higher mortality with treatment delay after blood culture draw, while Kollef et al. extended this observation to patients with candidemia-associated septic shock and also emphasized the importance of source control for outcome [7, 11, 17]. In a later multicenter study including 216 patients from five European centers, Bassetti et al., in patients with septic shock due to candidemia, found that inadequate antifungal therapy was associated with mortality, together with inadequate source control and higher APACHE II score. However, they did not observe a significant association between mortality and the time from positive blood culture to initiation of adequate antifungal therapy [30]. More recently, Zhang et al. also reported a positive association between empirical antifungal therapy and survival in ICU patients with invasive fungal infection, although this study was not restricted to BSI [13]. In contrast, several other studies did not confirm a clear survival benefit of earlier antifungal treatment, particularly in ICU populations. Charles et al. found no improvement in outcome with therapy initiated within the first 2 days in ICU patients with candidemia [31]. Marriott et al. similarly reported that mortality in ICU candidemia was more strongly related to host factors than to treatment variables [15]. In hospitalized patients with candidemia, Kludze-Forson et al. and Grim et al. also did not report a significant association between timing of appropriate antifungal therapy and mortality [6, 14]. This absence of clear mortality benefit was further supported by the later studies of González-Lara et al. and Ohki et al. [16, 32]. Comparisons across studies are nevertheless limited by non-uniform definitions of time to therapy, with time zero variably defined as blood culture sampling, blood culture positivity, or not explicitly reported. This issue is particularly relevant in candidemia, where blood culture time to positivity may vary and reflect fungal burden.

In our international study, we found no association between early adequate antifungal therapy and mortality. As shorter blood culture time to positivity may reflect higher fungal burden, we performed a sensitivity analysis excluding patients with time to positivity within two days. The findings remained consistent, making it less likely that the absence of an observed mortality benefit was explained by patients with rapid culture positivity.

Across studies, including ours, illness severity, as reflected by the SOFA score, appears to be a major driver of mortality in critically ill patients with candidemia, which may partly explain why earlier antifungal therapy has not consistently shown a survival benefit [2, 6, 11, 16]. Baseline imbalances between treatment groups also suggest that empirical antifungal therapy was influenced by clinical severity and suspicion of candidemia. Residual confounding by indication may therefore persist due to unmeasured prognostic factors influencing treatment decisions. Another possible explanation is that documented candidemia may represent a relatively late stage of invasive Candida infection, particularly in non-catheter-related disease, potentially limiting the impact of antifungal therapy initiated after BSI onset. In our study, failure to achieve source control was also associated with higher 28-day mortality, consistent with previous EUROBACT data and candidemia studies emphasizing the prognostic importance of adequate source control [24, 33]. Importantly, source control was achieved in most patients when indicated, particularly through catheter removal in catheter-associated episodes, supporting its central role in the management of candidemia. Our study adds further information by focusing specifically on ICU candida BSI and by including patients from both EUROBACT-1 and EUROBACT-2, thereby providing a large international cohort across all continents and multiple centers. It also extends prior evidence by including a more contemporary population and by showing that the absence of a significant association between early adequate antifungal therapy and mortality was consistent across time, despite possible changes in practice and antifungal management over the last decade.

By focusing specifically on the first 2 days after blood culture draw, our study assessed a clinically relevant and usable treatment window. Although our study is based on observational data, these findings may inform the use of empirical antifungal therapy during the period before candidemia is confirmed. In ICU practice, antifungals are often started empirically in severely ill patients. However, antifungal exposure in the ICU has been shown to be frequently unnecessary [10]. In a multicenter cross-sectional study, antifungal therapy was administered to 7.5% of ICU patients, although approximately two-thirds eventually had no documented invasive fungal infection [34]. Similarly, in the AmarCAND2 study, invasive candidiasis was ultimately proven in only 48% of ICU patients receiving systemic antifungal therapy for suspected or proven infection, highlighting the diagnostic uncertainty surrounding empirical antifungal treatment [35]. This is particularly relevant as antifungal use is associated with drug toxicity, emergence of resistance, selection of non-albicans species and higher costs [10].

Our results suggested that an early strategy may not benefit all ICU patients with candidemia and that severity of illness and source control remain the principal determinants of outcome. Rather than supporting broad pre-emptive antifungal treatment before candidemia is documented, our findings support better patient selection and a more individualized approach, integrating clinical severity and the likelihood of invasive Candida infection. Further studies are needed to identify the subgroup of patients who may still require immediate empirical treatment and to confirm these findings prospectively.

Some study limitations should be acknowledged. First, the observational design precludes any causal interpretation, however, we applied complementary statistical methods to mitigate risk of confounding bias. Nevertheless, unmeasured factors influencing treatment decisions may have resulted in residual confounding by indication. Second, although this observational study did not show a significant association, limited statistical power cannot be excluded; however, it represents one of the largest cohorts on this topic and includes data from ICUs worldwide. Third, treatment duration was not assessed, although it may have influenced patient outcomes. In addition, early adequate antifungal therapy was defined within a 2 days window after blood culture draw as a pragmatic exposure definition that may not fully capture differences in the exact timing of treatment initiation within this interval. However, similar definitions have commonly been used in previous studies on this topic. Fourth, EUROBACT-1 and − 2 were not specifically designed for candidemia, and antifungal management was not standardized across centers. Data on the use of candidemia-specific risk scores or rapid diagnostic tests to guide empirical antifungal therapy were not available. However, the international multicenter design captures real-world practice across diverse ICU settings, and results were consistent across study periods. Finally, patients who died within the first 2 days after blood culture draw were excluded to reduce immortal time bias. Although this may limit generalizability to the most acute presentations, the overall 28-day mortality in our cohort was approximately 40%, in line with previous reports in critically ill patients with candidemia.

## Conclusions

In this secondary analysis of EUROBACT-1 and EUROBACT-2, early adequate antifungal therapy was not significantly associated with improved survival. These results challenge the unselected empirical use of antifungal treatment while awaiting blood culture results, especially in view of the potential toxicity and stewardship concerns associated with unnecessary antifungal exposure. Further studies are needed to identify the subgroup of patients who may still require immediate empirical treatment and to confirm these findings prospectively.

## Supplementary Information

Below is the link to the electronic supplementary material.

Supplementary Material 1

## Data Availability

The datasets used and/or analyzed during the current study are available from the corresponding author on reasonable request.
