# Supplementary material for: Early adequate empiric antifungal therapy and 28-day mortality in ICU patients with candidemia: a pooled patient-level analysis of EUROBACT-1 and EUROBACT-2 cohorts
Source: Crit Care. 2026 Oct 16;30:571. doi: 10.1186/s13054-026-06373-3 (PMC13741774; doi:10.1186/s13054-026-06373-3)
Supplement: Supplementary file 1 [file 13054_2026_6373_MOESM1_ESM.docx]

**Supplementary figures and tables**

**Supplementary Figure 1. directed acyclic graph (DAG)**

Legend: Directed acyclic graph illustrating the assumed relationships between early adequate antifungal therapy, 28-day mortality, and relevant clinical covariates.

**Supplementary Table 1. Description of the 18 patients excluded because of death within the first 2 days**

| **Patient** | **Age (years)** | **Sex** | ***Candida* species** | **Treated** | **If treated** |
| --- | --- | --- | --- | --- | --- |
| 1 | 86 | Male | *Candida albicans* | No |  |
| 2 | 56 | Male | *Candida auris* | No |  |
| 3 | 54 | Female | *Candida sp.* | Yes | fluconazole |
| 4 | 78 | Female | *Candida parapsilosis complex* | No |  |
| 5 | 79 | Female | *Candida albicans* | No |  |
| 6 | 60 | Male | *Candida parapsilosis complex* | Yes | anidulafungin |
| 7 | 57 | Female | *Candida parapsilosis complex* | No |  |
| 8 | 82 | Female | *Candida albicans* | Yes | micafungin |
| 9 | 31 | Male | *Candida krusei* | No |  |
| 10 | 69 | Male | *Candida parapsilosis complex* | Yes | micafungin |
| 11 | 60 | Female | *Candida tropicalis* | No |  |
| 12 | 60 | Male | *Candida albicans* | No |  |
| 13 | 57 | Male | *Other Candida species* | Yes | caspofungin |
| 14 | 44 | Female | *Candida parapsilosis complex* | No |  |
| 15 | 32 | Male | *Candida albicans* | Yes | fluconazole |
| 16 | 26 | Male | *Candida albicans* | Yes | amphotericin B |
| 17 | 82 | Male | *Candida albicans* | No |  |
| 18 | 81 | Male | *Candida albicans* | Yes | fluconazole |

**Supplementary Table 2: Center-level ICU characteristics and organization**

| **158 centers** | |
| --- | --- |
| **Country** | **N (%)** |
| Australia | 6 (3.80%) |
| Bangladesh | 1 (0.63%) |
| Belgium | 3 (1.90%) |
| Brazil | 2 (1.27%) |
| Brunei | 1 (0.63%) |
| Canada | 3 (1.90%) |
| China | 5 (3.16%) |
| Colombia | 1 (0.63%) |
| Dubai | 1 (0.63%) |
| Egypt | 1 (0.63%) |
| France | 18 (11.39%) |
| Germany | 5 (3.16%) |
| Greece | 19 (12.03%) |
| Hong Kong | 1 (0.63%) |
| India | 3 (1.90%) |
| Iran | 1 (0.63%) |
| Israel | 2 (1.27%) |
| Italy | 6 (3.80%) |
| Japan | 2 (1.27%) |
| Kazakhstan | 1 (0.63%) |
| Lebanon | 1 (0.63%) |
| Libya | 1 (0.63%) |
| Malaysia | 3 (1.90%) |
| Netherlands | 3 (1.90%) |
| Nigeria | 1 (0.63%) |
| Poland | 1 (0.63%) |
| Portugal | 7 (4.43%) |
| Republic of Ireland | 1 (0.63%) |
| Republic of Korea | 4 (2.53%) |
| Romania | 2 (1.27%) |
| Russian Federation | 1 (0.63%) |
| Saudi Arabia | 1 (0.63%) |
| Singapore | 1 (0.63%) |
| Spain | 13 (8.23%) |
| Switzerland | 2 (1.27%) |
| Taiwan | 3 (1.90%) |
| Thailand | 2 (1.27%) |
| Turkey | 21 (13.29%) |
| UK | 8 (5.06%) |
| **Hospital funding** | |
| Public | 135 (85.44%) |
| Private | 23 (14.56%) |
| **ICU Type** | |
| Medical | 26 (16.46%) |
| Surgical | 12 (7.59%) |
| Mixed | 120 (75.95%) |
| **ICU Structure** | |
| Open | 34 (21.52%) |
| Closed | 124 (78.48%) |
| **Antibiotic stewardship and pharmacological support available** | |
| No | 43 (27.22%) |
| Yes | 115 (72.78%) |
| **Infectious disease specialist availability** | |
| No | 12 (7.59%) |
| Yes | 139 (87.97%) |
| Missing | 7 (4.43%) |

*^1^Defined based on the availability of aminoglycoside therapeutic drug monitoring (TDM)*

**Supplementary Figure 2. Details on the time to adequate treatment**

Legend: Distribution of patients according to time from bloodstream infection (BSI) onset to initiation of adequate antifungal therapy. Patients were categorized as receiving adequate therapy within ≤1d, >1 to ≤2 d, >2 to ≤5d, or >5d/never.

**Supplementary Figure 3. Love plot showing covariate balance before and after inverse probability of treatment weighting (IPTW)**

Legend: Standardized mean (SMD) differences are presented for each baseline covariate included in the propensity score model, comparing patients who received early adequate antifungal therapy within 2 days with those who did not. Balance was assessed before and after weighting, with smaller absolute standardized mean differences indicating better covariate balance.

**Supplementary Figure 4. Kaplan-Meier survival estimates**

Legend: Kaplan–Meier curves showing 28-day survival in patients who received early adequate empirical antifungal therapy within 2 days after blood culture draw compared with those who did not.

**Supplementary Table 3. Univariable and multivariable frailty Cox models assessing the association between early adequate antifungal therapy and 28-day mortality**

| **Variable** | **HR (95% CI)** | **p-value** |
| --- | --- | --- |
| **Univariable frailty Cox model** |  |  |
| Empiric adequate antifungal <=2d (yes vs no) | 1.27 (0.87–1.88) | 0.219 |
| **Multivariable frailty Cox model** |  |  |
| Empiric adequate antifungal <=2d (yes vs no) | 1.29 (0.86–1.93) | 0.227 |
| *Source control adequacy*  -not needed -required and complete -required but not achieved | Reference  1.03 (0.58–1.83)  2.11 (1.09–4.09) | 0.913  0.026 |
| *Source of infection*  Other  Catheter  Abdominal | Reference  1.01 (0.57–1.79)  0.82 (0.43–1.56) | 0.966  0.540 |
| Antibiotic stewardship and pharmacological support available | 0.97 (0.64–1.48) | 0.895 |
| SOFA score | 1.13 (1.07–1.18) | <0.001 |
| Charlson comorbidity index | 1.05 (0.97–1.14) | 0.222 |
| Admission type: Surgical (reference: medical) | 0.70 (0.44–1.12) | 0.139 |

Multivariable model adjusted for source of infection, therapeutic drug monitoring of aminoglycosides, SOFA score, Charlson comorbidity index and admission type. HR: hazard ratio; CI: confidence interval; SOFA: Sequential Organ Failure Assessment.

**Supplementary Table 4. Subgroup analyses using weighted Cox regression for 28-day mortality, stratified by study cohort, hypotension status, Candida species and time to positivity**

| **Eurobact-1, N=81** | **HR (95% CI)** | **p** |
| --- | --- | --- |
| Weighted Cox model (IPTW) |  |  |
| Empiric adequate antifungal <=2d (yes vs no) | 1.26 (0.57-2.79) | 0.564 |
| **Eurobact-2, N=191** | **HR (95% CI)** | **p** |
| Weighted Cox model (IPTW) |  |  |
| Empiric adequate antifungal <=2d (yes vs no) | 1.25 (0.78-1.99) | 0.347 |
| **Candida albicans only, N=128** | **HR (95% CI)** | **p** |
| Weighted Cox model (IPTW) |  |  |
| Empiric adequate antifungal <=2d (yes vs no) | 1.42 (0.79-2.54) | 0.243 |
| **Other Candida species, N=144** | **HR (95% CI)** | **p** |
| Weighted Cox model (IPTW) |  |  |
| Empiric adequate antifungal <=2d (yes vs no) | 1.07 (0.62-1.87) | 0.799 |
| **Candida glabrata only, N=47** | **HR (95% CI)** | **p** |
| Weighted Cox model (IPTW) |  |  |
| Empiric adequate antifungal <=2d (yes vs no) | 0.44 (0.15-1.29) | 0.137 |
| **Other Candida species, N=225** | **HR (95% CI)** | **p** |
| Weighted Cox model (IPTW) |  |  |
| Empiric adequate antifungal <=2d (yes vs no) | 1.44 (0.93-2.22) | 0.103 |
| **Hypotension* at BSI, N=108** | **HR (95% CI)** | **p** |
| Weighted Cox model (IPTW) |  |  |
| Empiric adequate antifungal <=2d (yes vs no) | 1.58 (0.89-2.81) | 0.116 |
| **Absence of hypotension* at BSI, N=146** | **HR (95% CI)** | **p** |
| Weighted Cox model (IPTW) |  |  |
| Empiric adequate antifungal <=2d (yes vs no) | 1.03 (0.57-1.85) | 0.923 |
| **Time to positivity >=2 days^**^** | **HR (95% CI)** | **p** |
| Weighted Cox model (IPTW) |  |  |
| Empiric adequate antifungal <=2d (yes vs no) | 1.09 (0.62-1.90) | 0.771 |

*18 missing

** Patients with a missing date of BSI positivity (n=52) or time to positivity <2 days (n=71) were excluded.

**Supplementary Table 5. Sensitivity analysis using a 1-day definition of early adequate antifungal therapy**

|  | **HR (95% CI)** | **p-value** |
| --- | --- | --- |
| **Univariable Cox model** |  |  |
| Empiric adequate antifungal <=1d (yes vs no) | 1.27 (0.81-2.00) | 0.306 |
| **Multivariable Cox model^1^** |  |  |
| Empiric adequate antifungal <=1d (yes vs no) | 1.32 (0.85-2.04) | 0.216 |

^1^Adjusted for source of infection, source control management, availability of antimicrobial stewardship/pharmacological support, SOFA score at BSI, Charlson Comorbidity Index, admission type, and mechanical ventilation at the time of BSI.
